# Evaluation of Two Methods (Inside-Out/Outside-In) Inferior Articular Process Resection for Uniportal Full Endoscopic Posterolateral Transforaminal Lumbar Interbody Fusion: Technical Note

**DOI:** 10.3390/brainsci11091169

**Published:** 2021-09-03

**Authors:** Hyeun-Sung Kim, Pang-Hung Wu, Jin-Woo An, Yeon-Jin Lee, Jun-Hyung Lee, Myeong-Hun Kim, Inkyung Lee, Jong-Sung Park, Jun-Hyung Lee, Jun-Hwan Park, Il-Tae Jang

**Affiliations:** 1 Spine Surgery, Nanoori Gangnam Hospital, Seoul 06048, Korea; nigaheboa@gnnanoori.co.kr (Y.-J.L.); drbrainlee@gmail.com (J.-H.L.); michaelkmh@hanmail.net (M.-H.K.); ikl84@naver.com (I.L.); Siren7317@naver.com (J.-S.P.); nanooriresearch@gmail.com (I.-T.J.); 2 Orthopaedic Surgery, JurongHealth Campus, National University Health System, Singapore 609606, Singapore; 3 Nanoori Hospital Spine And Joints Clinic, Dubai 66566, United Arab Emirates; drbear94@gmail.com; 4 Department of Internal Medicine, Chosun University School of Medicine, Gwangju 61452, Korea; pp3614@naver.com; 5 Neurosurgery Department, Medical School University, 4032 Debrecen, Hungary; yyea7133@gmail.com

**Keywords:** endoscopic spine surgery, transforaminal lumbar interbody fusion, degenerative spine disease, endoscopic lumbar interbody fusion, spinal fusion, minimally invasive spine surgery

## Abstract

**Objective:** There is limited literature comparing the uniportal full endoscopic posterolateral transforaminal lumbar interbody fusion outside-in approach (ETLIF (O)) with the inside-out approach (ETLIF (I)). **Methods:** Radiological evaluation was performed on disc height restoration and coronal wedging angle, and operation time (inferior articular process resection time/total operation time) and clinical evaluation were made. **Result:** 48 cases of inside-out and 38 cases of outside-in cases were included. Compared to inside-out, the outside-in approach had significantly less operative time required to resect inferior articular process: 36.55 ± 10.37, and total operative time: 87.45 ± 20.14 min compared to 49.83 ± 23.97 and 102.56 ± 36.53 min, respectively, for the inside-out approach, *p* < 0.05. Compared to the preoperative state, both cohorts achieved significant improvement of VAS and ODI at post-operative 1 week, 3 months and at final follow up. Both cohorts achieved statistically significant increased disc height with 5.00 ± 2.87 mm, 5.49 ± 2.33 mm and statistically significant improvement in coronal wedge angle with 1.76 ± 1.63°, 3.24 ± 2.92° in the inside-out and outside-in approaches respectively. **Conclusions:** Complete removal of inferior articular process is the key part of endoscopic fusion with two methods that can be applied: an inside-out approach or an outside-in approach. Comparing both techniques, the outside-in approach has a shorter operative time required for inferior articular process resection and total length of operation with similar good clinical and radiological outcomes.

## 1. Introduction

With the evolution of endoscopic spine surgery, there are an increasing variety of endoscopic techniques being applied in lumbar spine surgery [1,2]. Both transforaminal and interlaminar approaches have been described to treat various degenerative conditions of the lumbar spine [3,4,5,6,7]. Endoscopic lumbar interbody fusion through the transforaminal route in Kambin’s triangle (KLIF) has a relatively longer history than endoscopic lumbar interbody fusion through the posterolateral route (ETLIF). KLIF is a popular fusion technique performed under local anesthesia with monitored sedation; it tends to preserve the lumbar facet in the process of fusion surgery. However, there is a higher risk of subsidence rate and exit nerve root injury. There is no direct decompression in KLIF and additional decompression is required if there is significant spinal stenosis. As the space in Kambin’s triangle is limited, there is a tendency to use a small width footprint cage for KLIF [8,9,10,11]. Facet sacrificing transforaminal lumbar interbody fusion is a good standard popular technique in the open, minimally invasive tubular approach and is recently becoming more popular in biportal endoscopic assisted fusion [12,13,14,15]. However, there is limited literature on uniportal endoscopic posterolateral lumbar interbody fusion with complete facet resection [16]. Kim and Wu et al. described the uniportal full endoscopic approach to perform posterolateral transforaminal lumbar interbody fusion (ETLIF) with facet resection in grade 2 spondylolisthesis, scoliosis of less than 30° curve and severe foraminal stenosis patients [17,18,19]. Further evolution of the ETLIF technique raises a question of whether there would be a difference in removing the inferior articular process (IAP) from an inside-out approach as compared to an outside-in approach. There is only one study we found that described the outside-in approach for ETLIF [20]. In this study, we performed a retrospective comparative cohort study to evaluate the operative, radiological roentgenogram outcomes and clinical outcomes of ETLIF inside-out, ETLIF (I) as compared to outside-in and ETLIF (O) for single level lumbar interbody fusion.

## 2. Materials and Methods

### 2.1. Indication, Inclusion and Exclusion Criteria

Informed consent was obtained from all patients that participated in this retrospective comparative study and was reviewed by Institutional review board of Nanoori Hospital, Seoul, Republic of Korea (NR-IRB 2021-004).

The inclusion criteria were patients who presented with neurogenic claudication and back pain who had failed the minimum 6 weeks of conservative treatment and had one of the following diagnoses: (1) spinal stenosis with instability of lumbar segment; (2) grade 2 and below spondylolisthesis; (3) foraminal stenosis; or (4) Recurrent disc herniation. Each patient had a single level uniportal endoscopic posterolateral lumbar interbody fusion (ETLIF). The inferior articular process resections were carried out in two distinct approaches. In the uniportal endoscopic posterolateral lumbar interbody fusion using inside-out approach, ETLIF (I), the inferior articular process (IAP) was resected starting from the spinolaminar junction outwards towards the lateral laminofacet junction [17,18,19]. In the uniportal endoscopic posterolateral lumbar interbody fusion using outside-in approach, ETLIF (O), IAP was resected starting from the lateral laminofacet junction inwards towards the spinolaminar junction [20]. We evaluate the differences between the two techniques in terms of intraoperative and postoperative parameters.

We excluded patients who had spinal fusion surgery due to revision spinal fusion surgery, trauma, tumor, pseduoarthrosis, infection, congenital spinal deformity, sagittal malalignment and coronal malalignment with more than 10 degrees coronal curve.

### 2.2. Endoscopic Surgical Anatomy of Inferior Articular Process in Endoscopic Fusion

**Figure 1:**
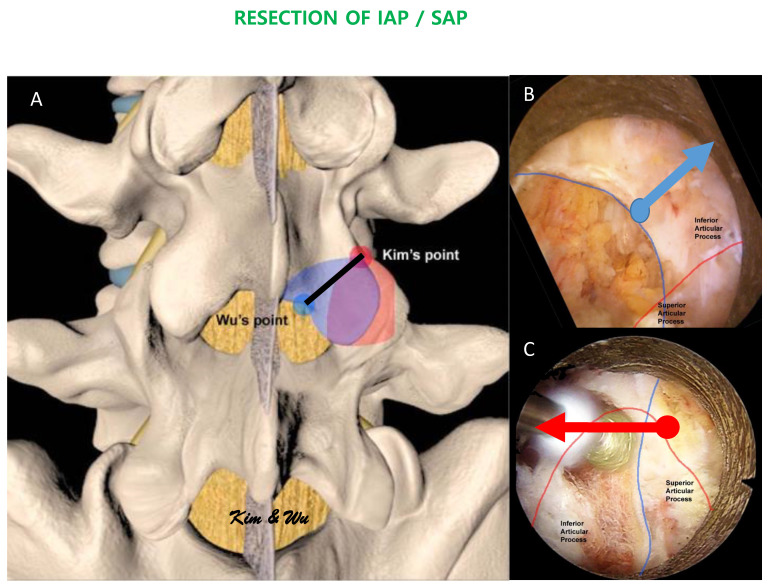
(**A**): Illustration of the margin of inferior articular process. The endoscopic inferior articular facet resection required is marked by a black line. Wu’s point (blue circle) is at the mid-point of the bony arch which forms from the ipsilateral spinolaminar junction of the cephalad lamina to the most inferomedial rounded edge of the inferior articular process. Kim’s point is the confluence of the superolateral edge of the inferior articular facet and the superolateral edge of the superior articular facet. The amount of recommended inferior articular process resection is shaded in blue while the amount of recommended superior articular facet resection is shaded in red. (**B**): In uniportal endoscopic posterolateral lumbar interbody fusion Inside-Out Approach, ETLIF (I), an oblique upwards and lateral direction endoscopic drilling is carried out from Wu’s point towards Kim’s point. (**C**): In uniportal endoscopic posterolateral lumbar interbody fusion Outside-in Approach, ETLIF (O), an oblique medial inferior endoscopic drilling is carried out from Kim’s point to Wu’s point.

Inferior articular process is a process of a cephalad vertebra of the spinal segment that lies on each side of the neural arch and projects downward and processes vertical convex articular facets which face anterolaterally articulating with the superior articular process of the next caudal vertebra. It is convex dorsally and laterally (Figure 1A) [21]. In the magnified field under endoscope, the superomedial aspect of the inferior articular facet is often medial and deep to the mid-point of the bony arch forms from the ipsilateral spinolaminar junction of the cephalad lamina to the most inferomedial rounded edge of the inferior articular process, which we labelled as Wu’s point in this paper for illustration purposes (Figure 1A,B). This point is at the sloping edge of the medial laminofacet junction medial to which the inferior articular process slopes ventrally caudally and laterally to join the medial superior edge of the superior articular process. The rotatores spinae muscles are associated medial to the edge. Profuse bleeding can occur leading to poor visualization of the endoscopic view when soft tissue dissection occurs medial to the edge of Wu’s point. From Wu’s point, the endoscope moves obliquely upwards and laterally on the dorsal convexity of the inferior articular process to the superolateral edge of the inferior articular facet which articulates the superolateral edge of the superior articular facet, which we have labelled as Kim’s point for illustration purposes. Kim’s point is therefore the confluence of the superolateral edge of the superior articular process and the superolateral edge of inferior articular process (Figure 1A,C). The Kim’s point is often covered with facet capsule. Exposure of Kim’s point as an endoscopic anatomical landmark involves using a flexible curve radiofrequency ablator to dissect the facet capsule on the lateral edge of the facet joint, followed by endoscopic drilling at the lower and lateral edges of the inferior articular facet to expose the underlying lateral edge of the superior articular facet. Once the superior articular facet was found, we continued endoscopic drilling in the cranial direction along the exposed facet joint to the superolateral edge of the facet joint, which would be Kim’s point. The multifidus muscle drapes this bony region, lateral to Kim’s point is the intertransverse membrane and ventral to the intertransverse membrane is associated with radicular artery and exiting nerve root (care is taken during endoscopic soft tissue dissection lateral to Kim’s point, so as not to breach the intertransverse membrane and cause unnecessary bleeding of the radicular artery).

### 2.3. Surgical Technique of ETLIF

Uniportal endoscopic posterolateral lumbar interbody fusion, ETLIF technique has been described [17,18,19,20]. Further evolution of the technique has divided the approach to inside-out approach, ETLIF (I) and outside-in approach, ETLIF (O).

### 2.4. Surgical Procedure for ETLIF (I) and ETLIF (O)

#### 2.4.1. Docking of Endoscope on Isthmus

**Figure 2:**
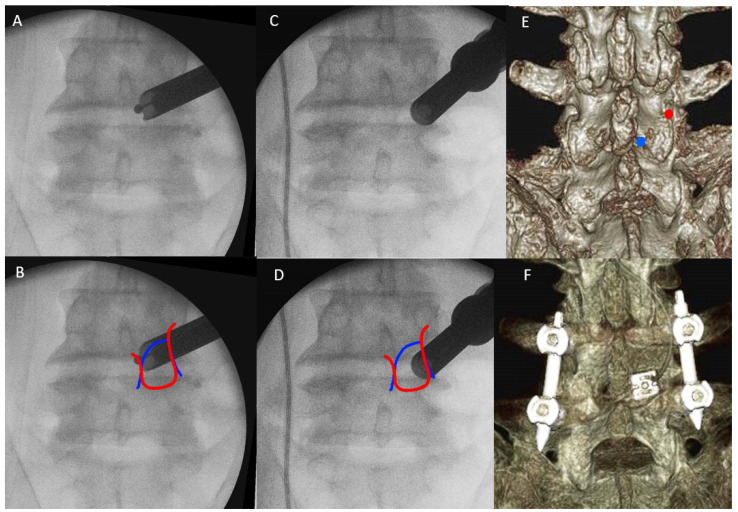
(**A**) The endoscope is docked and moved to Wu’s point. (**B**) Wu’s point is close to the fluoroscopic image of the medial confluence of medial edge of outline of inferior articular process (red line) and superior articular process (blue line). (**C**) The endoscope is docked and moved to Kim’s point. (**D**) Kim’s point is close to the fluoroscopic image of the lateral confluence of lateral edge of outline of inferior articular process (red line) and superior articular process (blue line). (**E**) Preoperative 3D reconstructed CT scan assisted the planning of docking region, on Wu’s point (blue circle) and Kim’s point (red circle). (**F**) Postoperative 3D reconstructed CT showed the final position of the cage and pedicle screws.

The patient was positioned prone on a Wilson Frame on top of a radiolucent operating table with the spine in slight flexion under general anesthesia. The Wilson Frame was flattened during pedicle screws and rod insertion for reduction maneuvers. We performed facet resection and cage insertion from the patient’s symptomatic side. Cage insertion was done before pedicle screws insertion in all cases. We used a 1.6 cm skin incision on the cephalad vertebral pedicle of the symptomatic side and level for facet resection, disc preparation and cage insertion. A wider 3 cm fascia incision was made for more mobility of the endoscope. Serial dilation with guidewire, obturators and finally a working retractor cannula was docked on the medial part of the inferior border of IAP. After docking to the safe area, we moved the endoscope to Wu’s point or Kim’s point of the cephalad vertebra for ETLIF (I) and ETLIF (O), respectively (Figure 2). We used a 13.7 mm outer diameter beveled tip working retractor cannula. We performed an intraoperative anteroposterior and lateral view at this point of time to confirm the correct level of interbody fusion. We then inserted a 15^0^ viewing angle, 10 mm outer diameter, 6 mm working channel diameter and 125 mm working length endoscope to begin the surgical procedure.

#### 2.4.2. Handling of Inferior Articular Process: ETLIF (I) versus ETLIF (O)

**Figure 3:**
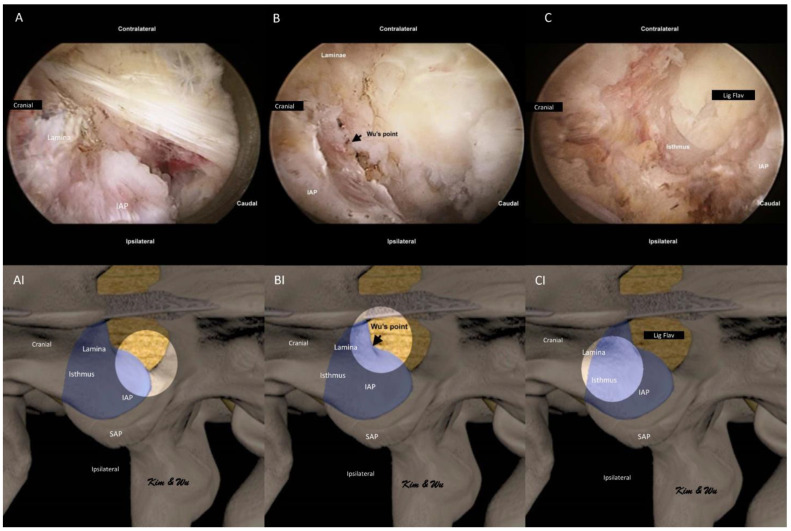
Endoscopic pictures of the uniportal endoscopic posterolateral lumbar interbody fusion Inside-Out Approach, ETLIF (I) handling of inferior articular process of left L4/5. (**A**) Soft tissue dissection of the multifidus muscle which is draped over the isthmus and inferior articular process (IAP). Exposure of the medial edge of the lamina, spinolaminar junction and round medial edge of IAP. (**AI**) Illustration of anatomical structures of (**A**), with the bright white circle highlighting the focus region of endoscopic anatomy in corresponding (**A**) at the rounded medial edge of IAP. (**B**) Exposure of Wu’s point, the midpoint of the bony arch formed from the spinolaminar junction and rounded inferior medial edge of the inferior articular facet. (**BI**) Illustration of anatomical structures of (**B**), with the bright white circle highlighting the focus region of endoscopic anatomy in corresponding (**B**) at the Wu’s point. (**C**) Endoscopic drilling done layer by layer to the inner layer of cortex of isthmus, thinning the isthmus to inner cortex. (**CI**) Illustration of anatomical structures of (**C**), with the bright white circle highlighting the focus region of endoscopic anatomy in corresponding (**C**) at the isthmus of left L4.

We used normal saline with an irrigation pressure of 25–40 mm Hg with an irrigation pump during most parts of the endoscopic fusion procedure. We first performed soft tissue dissection around the medial aspect of the facet joint, the medial edge of laminar and isthmus with radiofrequency ablator. We looked for the endoscopic landmark of the spinolaminar junction and medial rounded edge of the facet joint (Figure 3A,AI). We dissected the soft tissue and identified the midpoint of the bony arch formed from the spinolaminar junction and the most inferomedial aspect of the inferior articular facet labelled as Wu’s point (Figure 3B,BI). Endoscopic drilling was started at Wu’s point for ETLIF (I). In ETLIF (I), resection of IAP was done starting from Wu’s point drilling of IAP in an oblique inside-out, medial to lateral and caudal to cephalad direction towards Kim’s point. During endoscopic drilling, the initial outer cortical bone zone of isthmus had minimal bleeding, which was followed by a bleeding cancellous bone zone which often required radiofrequency ablation for hemostasis and finally the inner cortical bone of isthmus which had dense bone with minimal bleeding. The inner cortical layer was drilled till a thin layer was left. Using the working retractor tube, we put direct pressure on the IAP which would perform a control fracture of the thin residual layer of IAP from the endoscopic drilling track. IAP was harvested as an autograft.

**Figure 4:**
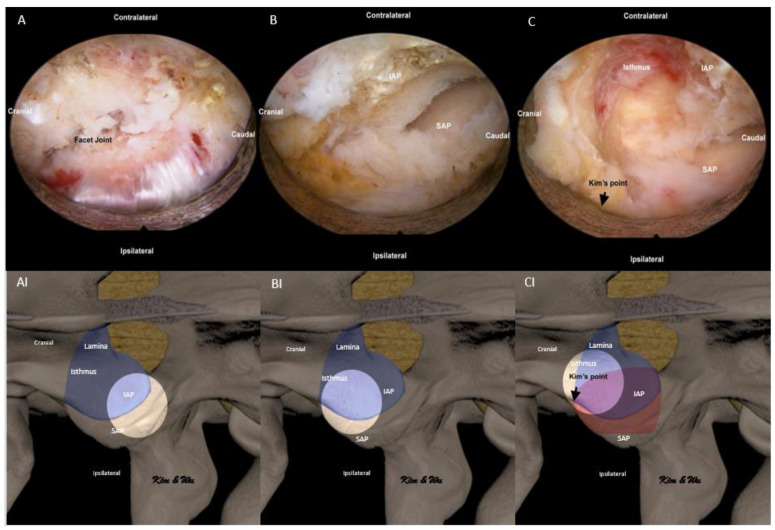
Endoscopic pictures of uniportal endoscopic posterolateral lumbar interbody fusion outside-in approach, ETLIF (O) handling of inferior articular process (IAP) of left L4/5. (**A**) Exposure of lateral aspect of facet joint with radiofrequency ablator. (**AI**) Illustration of anatomical structures of (**A**), with the bright white circle highlighting the focus region of endoscopic anatomy in corresponding (**A**) at the lateral aspect of the facet joint. (**B**) Radiofrequency ablation was applied to release the facet capsule and endoscopic drilling at the lower and lateral edges of the inferior articular facet to expose the underlying lateral edge of the superior articular facet (SAP). (**BI**) Illustration of anatomical structures of (**B**), with the bright white circle highlighting the focus region of endoscopic anatomy in corresponding (**B**) at the lateral aspect of IAP and SAP. (**C**) After identification of Kim’s point at the confluence of the superior lateral edge of IAP and superior articular process (SAP) of the facet joint, endoscopic drilling of isthmus is performed from lateral to medial and cephalad to caudal direction (outside-in). (**CI**) Illustration of anatomical structures of (**C**), with the bright white circle highlighting the focus region of endoscopic anatomy in corresponding (**C**) at the superolateral aspect of the facet joint (Kim’s point) and lateral aspect of isthmus.

**Figure 5:**
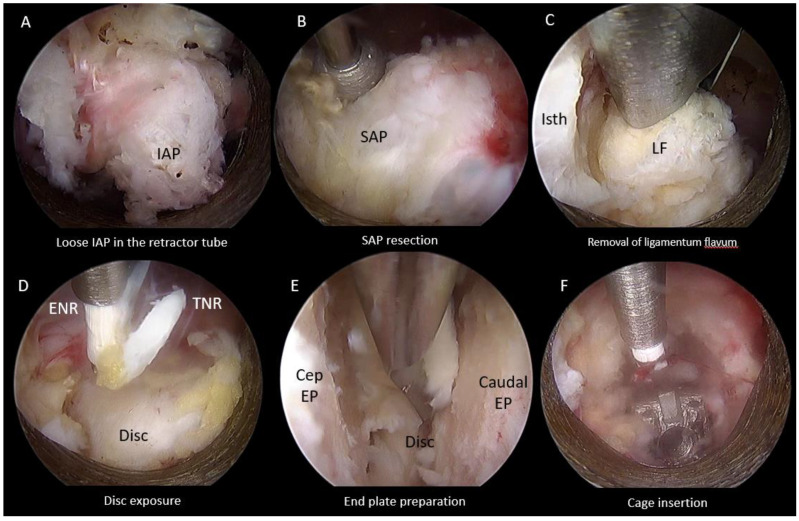
Endoscopic pictures of uniportal endoscopic posterolateral lumbar interbody fusion procedure after inferior articular facetectomy of left L4/5. (**A**) Endoscopic Inferior Facetectomy completed with loose inferior articular process (IAP) isolated within retractor tube and retrieved as autograft for fusion. (**B**) Endoscopic Superior Facetectomy performed with endoscopic drill. (**C**) Ligamentum flavum removed with Kerrison Rongeur. (**D**) Traversing nerve root (TNR) and exiting nerve root (ENR) exposed after ligamentum flavum removed, hemostasis of epidural vessels on the disc performed. (**E**) Denudation of end plate cartilages from cephalad end plate (Cep EP) and caudal endplate (Caudal EP) with endoscopic drill, blunt bent dissector and pituitary forceps to remove disc and cartilage. (**F**) Interbody cage was inserted and inspected to be in optimal position in relation to end plates and neural elements.

In ETLIF (O), the abovementioned steps for handling of IAP were done in the reverse manner. Endoscopic soft tissue dissection of multifidus was done with focus on the lateral aspect of the facet joint (Figure 4A,AI). In order to identify endoscopic anatomical landmarks, at Kim’s point where the superolateral aspect of the inferior articular process intersects with the superolateral aspect of the superior articular process, radiofrequency ablation was applied to release the facet capsule and endoscopic drilling was carried out at the lower and lateral edges of the inferior articular facet to expose the underlying lateral edge of superior articular facet (Figure 4B,BI). Once the superior articular facet was found, we continued endoscopic drilling in the cranial direction along the exposed facet joint to the superolateral edge of the facet joint, labelled as Kim’s point (Figure 4C,CI). Often, the superior articular vessel lies in close proximity and hemostasis with radiofrequency ablator is necessary. From the identified Kim’s point, endoscopic drilling of the isthmus was carried out in an oblique outside-in, lateral to medial and cephalad to caudal direction towards Wu’s point in a layer by layer manner from outer cortex to inner cortex till a thin inner cortical layer was left. We extracted IAP with a working retractor tube by putting direct pressure on the IAP and performed a control fracture of the thin residual layer of IAP from the endoscopic drilling track. IAP was harvested as an autograft (Figure 5A).

#### 2.4.3. Superior Articular Facet Resection and Flavectomy

We followed resection of inferior articular process which was isolated within the retractor tube and removed as an autograft (Figure 5A). Superior articular facetectomy was performed as the next step of the procedure, from medial to lateral direction using an endoscopic drill (Figure 5B). Superior articular facet bone was harvested as autograft. We exposed the margins of the ligamentum flavum by carefully drilling the ipsilateral cranial lamina adjacent to the isthmus followed by caudal lamina to the margin of the ligamentum flavum and removed the ligamentum flavum (Figure 5C). When there was bilateral spinal stenosis, we performed over the top decompression of the contralateral side in some selected cases. Bony decompression was performed before ligamentum flavum removed. The dura sac and neural element were assessed on both ipsilateral and contralateral side for adequacy of decompression. Epidural bleeding was controlled with radiofrequency ablation and exposure of disc performed (Figure 5D).

#### 2.4.4. End Plate Preparation and Cage Insertion

The open beveled working cannula of 13.7 mm outer diameter and 10.2 mm inner diameter and placed on the disc while pointing the open bevel away from the exiting and traversing nerves to protect the neural elements. Gentle retraction of the neural elements could be completed with the retractor tube. Radiofrequency ablator and a blunt probe was used to perform annulotomy. Disc preparation and denudation of the end plate cartilage was carried out with an endoscopic drill and blunt bent probe until punctate bleeding of the subchondral bone, and the disc was retrieved with forceps (Figure 5E). Upon satisfactory end plate preparation, advancement of the retractor working cannula was made into the dorsal third of the intervertebral disc space at this moment of time, the neural elements were retracted out of harm’s way and the endoscope was withdrawn with the working cannula in place. The next portion of the procedure was completed under fluoroscopic guidance. Autograft and allograft admixture was inserted to the intervertebral disc space. An appropriate size trial was inserted to determine the size of the cage for insertion and to compact the bone graft in the intervertebral disc space. An appropriate size cage packed with autograft was introduced through the same working cannula or a Harrison cage glider into the appropriate position under fluoroscopic guidance [19]. Next, we reintroduced the endoscope to assess the status of neural decompression and the position of the cage making fine adjustments to the cage position using a punch under direct endoscopic vision (Figure 5F). A drain was inserted anchored with a suture (the drain would be removed on post-operative day 1).

The Wilson frame was subsequently flattened after cage insertion, and percutaneous pedicle screws were inserted under fluoroscopic guidance in standard fashions with or without cement augmentation. We introduced two bent rods of appropriate length and lordosis through the percutaneous rod. We performed compression and final tightening of the set screws and closed the wound in layers.

### 2.5. Collection of Operative, Clinical and Radiological Data

Both cohorts of patients underwent single level ETLIF performed in the period of October 2018 to August 2020 by a senior surgeon. Both techniques were started in October 2018.

**Figure 6:**
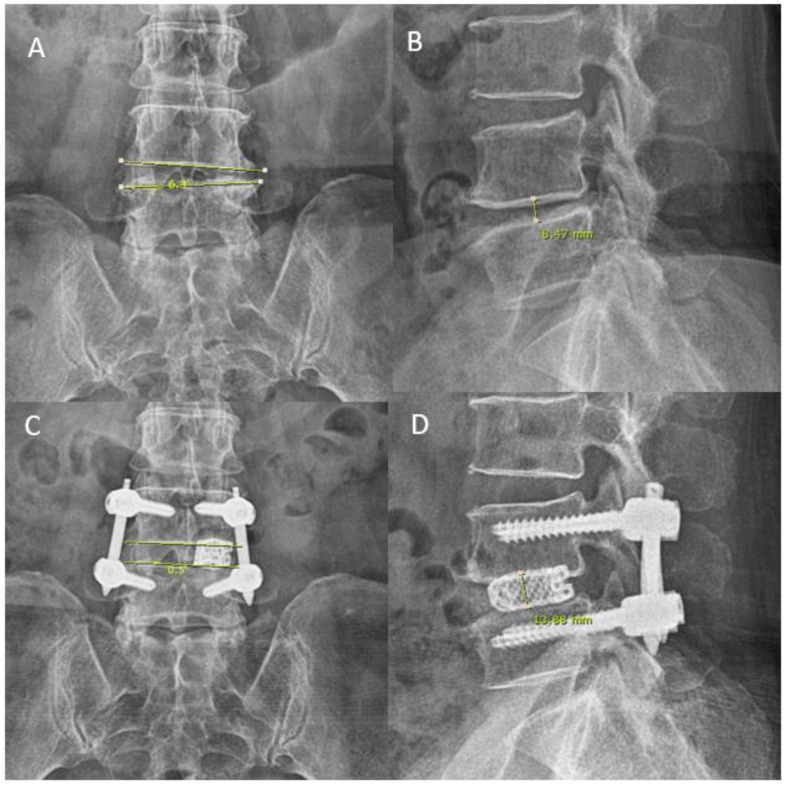
Roentgenogram measurement pre- and post-operative for disc height and coronal wedging in a 57 year old female who presented with pain score VAS 7 on the back with movement with bilateral lower limbs claudication. (**A**) Her preoperative anteroposterior roentgenogram measured a coronal wedge angle of 6.3°. (**B**) Her preoperative lateral roentgenogram measured a mid-disc height of 8.47 mm. (**C**,**D**) uniportal endoscopic posterolateral lumbar interbody fusion Outside-in Approach, ETLIF (O) performed, on postoperative day 1 roentgenogram, coronal wedge angle measured 0.5° anteroposterior roentgenogram and lateral roentgenogram measured mid-disc height of 13.88 mm.

We collected and analyzed baseline demographics data, operative data of inferior articular process resection time and total operative time, preoperative and postoperative radiographic roentgenogram data in disc height and coronal wedge angle (Figure 6). We measured clinical outcomes of Visual Analogue Scale and Oswestry Disability Index at preoperative, 1 week postoperative, 3 months postoperative and final follow up. MacNab’s criteria was evaluated at final follow up. X-ray was performed preoperatively, and on postoperative day one. Coronal wedge angle was measured as the angle subtended by line parallel to the cephalad end plate and caudal end plate. Mid disc height was measured in lateral view between midpoint of end plate of inferior border of cephalad vertebral body and midpoint of end plate of superior border of caudal vertebral body (Figure 6).

### 2.6. Statistical Analysis

Clinical data was analyzed with SPSS version 18 statistical analysis software (IBM corporation, New York, NY, USA). The continuous variables were expressed as mean and standard deviation (SD). The paired *t*-test was used for comparison of pre-operative and post-operative radiological roentgenogram results on disc height and coronal wedge angle. Clinical visual analogue scale (VAS) and Oswestry Disability Index (ODI) were measured at pre-operative, 4 weeks post-operative, 3 months post-operative and final follow up as well as MacNab’s score at final follow up reported by the patients were analyzed with paired *t* test. A value of (*p* < 0.05) was considered significant within each group of data. Independent T test was used to compare the clinical data of VAS and ODI and radiological roentgenogram results on disc height and coronal wedge angle.

## 3. Results

### 3.1. Baseline Demographics

From the period of October 2018 to August 2020, a total of 86 single levels ETLIF were performed in patients who met the inclusion and exclusion criteria, of which 48 were ETLIF (I) and 38 were ETLIF (O). No statistical difference in mean age of 65.02 ± 9.69 years in ETLIF (I) and 68.39 ± 11.41 years in ETLIF (O). The mean follow up was 14.73 ± 5.33 months in ETLIF (I) and 11.58 ± 3.81 months in ETLIF (O), *p* < 0.05.

In terms of the lumbar level treated in ETLIF (I), two L2/3, four L3/4, thirty-five L4/5 and seven L5/S1 ETLIF (I) were treated. All patients underwent general anesthesia for surgery. Four levels were fused for spinal stenosis with instability, forty levels were fused for spondylolisthesis and four levels were fused for foraminal stenosis.

In terms of lumbar level treated in ETLIF (O), there were five L2/3, two L3/4, twenty- two L4/5 and nine L5/S1 ETLIF (O) treated. All patients underwent general anesthesia for surgery. Five levels were fused for spinal stenosis with instability, thirty levels were fused for spondylolisthesis, two levels were fused for foraminal stenosis and one level was fused for recurrent disc herniation. There was no statistical difference between the two cohorts in terms of levels of operation and indication of operation (Table 1).

### 3.2. Intraoperative Timing and Radiological Parameters

ETLIF (O) achieved a statistically shorter time for inferior articular process resection and total operation time of 36.55 ± 10.37 min and 87.45 ± 20.14 min, respectively, as compared to ETLIF (I) of 49.83 ± 23.97 min and 102.56 ± 36.53 min respectively, *p* < 0.05 (Table 1).

ETLIF (I) achieved a statistically significant improvement of disc height and coronal wedge angle with 5.00 ± 2.87 mm and 1.76 ± 1.63°, *p* < 0.05. ETLIF (O) achieved statistically significant improvement of disc height and coronal wedge angle with 5.49 ± 2.33 mm and 2.87 ± 2.25°, *p* < 0.05. Between the ETLIF (I) and ETLIF (O) cohort, both achieved comparable improvement of disc height and coronal wedge angle with no statistical difference. There was no implant malposition or loosening in both cohorts of patients (Table 2, Table 3 and Table 4).

### 3.3. Clinical Outcomes

In terms of complications for ETLIF (I), we had two complications (4%) with one retained drain tip which was removed under local anesthesia and one revision ETLIF (I) for adjacent segment disease of L3/4 one year after ETLIF (I) of L4/5 performed. Both had good McNab’s criteria outcome and had improvement of his preoperative symptoms without neurological sequelae. In terms of complications for ETLIF (O), we had one complication (2.6%) for an adjacent disease of L3/4 with prolapsed intervertebral disc one year after ETLIF (O) of L2/3, he was treated with transforaminal endoscopic lumbar discectomy and had done well postoperatively with good McNab’s criteria outcome and improvement of his preoperative symptoms without neurological sequelae. There were no neurological complications in both the ETLIF groups of patients.

In terms of clinical results for ETLIF (I) preoperative, 1 week post-operative, 3 months post-operative and final follow up, the Visual Analog Scale (VAS) score had the mean and standard deviation of 7.65 ± 1.25, 3.44 ± 0.85, 2.33 ± 0.88 and 1.83 ± 0.86, respectively. Compared to the preoperative state, there was statistically significant improvement in VAS score at 1 weeks, 3 months and final follow up: 4.21 ± 1.49, 5.31 ± 1.68 and 5.81 ± 1.61, *p* < 0.05. The preoperative, 1 week post-operative, 3 months post-operative and final follow up Oswestry Disability Index (ODI) had the mean and range of 74.38 ± 8.72, 33.50 ± 6.52, 26.25 ± 4.72 and 23.81 ± 4.85 respectively. Compared to the preoperative state, there was statistically significant improvement in ODI score at 1 weeks, 3 months and final follow up: 40.88 ± 11.23, 48.13 ± 10.86 and 50.56 ± 10.63, *p* < 0.05. In terms of MacNab’s criteria, 1 had fair, 26 had good and 21 patients had excellent scores with 97.9% good to excellent score (Table 1 and Table 2).

In terms of clinical results for ETLIF (O) preoperative, the 1 week post-operative, 3 months post-operative and final follow up Visual Analog Scale (VAS) score had the mean and standard deviation of 7.58 ± 1.31, 3.26 ± 0.64, 2.39 ± 0.82 and 2.16 ± 0.86 respectively. Compared to the preoperative state, there was statistically significant improvement in VAS score at 1 weeks, 3 months and final follow up: 4.32 ± 1.32, 5.18 ± 1.47 and 5.42 ± 1.48, *p* < 0.05. The preoperative, 1 week post-operative, 3 months post-operative and final follow up Oswestry Disability Index (ODI) had the mean and range of 73.21 ± 10.62, 32.63 ± 5.44, 27.16 ± 5.31 and 25.05 ± 5.52, respectively. Compared to the preoperative state, there was statistically significant improvement in ODI score at 1 weeks, 3 months and final follow up, 40.58 ± 10.17, 46.05 ± 10.80 and 48.16 ± 11.71, *p* < 0.05. In terms of MacNab’s criteria, 1 had fair, 27 had good and 10 patients had excellent scores with 97.4% good to excellent score (Table 1 and Table 3).

Comparing the clinical results of ETLIF (I) and ETLIF (O), there was no statistically significant difference in terms of VAS, ODI and change in disc height and change of coronal wedge angle. There was no statistically significant difference between the two cohorts in terms of percentage of McNab’s score in good and excellent outcomes (Table 4).

## 4. Discussion

The proposed benefits of lumbar endoscopic spine surgery in the treatment of degenerative spine conditions were similar to other minimally invasive procedures [1,22]. Studies showed less blood loss, shorter hospital admission, less soft tissue damage and potentially less wound related complications in endoscopic and microscopic tubular decompression than open decompression. Some recent studies suggested the trend of less complications in endoscopic surgical decompression [23,24].

Such benefits lead to an increasing trend in the number of endoscopic spine procedures performed and a corresponding increased volume of publications on endoscopic decompression and KLIF type of endoscopic fusion; there was paucity of literature in ETLIF [16,17,18,19,22,25].

The main difference between KLIF [10,26] and ETLIF [17,18,19] was facetectomy and direct decompression. In KLIF, the facet joint was largely preserved with limited foraminoplasty to provide space for interbody cage insertion. As a result of limited space in Kambin’s triangle, often an expandable cage or an expandable mesh cage is necessary for ease of insertion of cage to prevent exiting nerve root dysesthesia [10,27]. The advantage of this approach is the ability to perform this procedure under local anesthesia with sedation and the proposed benefits of structural preservation and stability. The main disadvantage is the need for additional interlaminar decompression as a separate procedure if severe spinal stenosis is not correctable by reduction of spondylolisthesis, in addition to other established disadvantages of limited small foot print cage with subsidence and exiting nerve root dysesthesia [8,9,10,26].

The attraction of putting a large interbody cage with less soft tissue damage and yet providing direct decompression of neural elements led to the development of ETLIF [17,18,19,20]. There is less limitation in the position of the cage due to wider working space in the extended Kambin’s triangle after removal of the facet joint. The traditional large bullet shape TLIF cage used in open and microscopic tubular TLIF can be used in ETLIF. The cage can be placed more centrally by retracting the traversing nerve roots more medially using the working cannula retractor tube. Some of the key criticisms of this form of technique are its steep learning curve and its corresponding long duration of operative time as compared to open or microscopic transforaminal lumbar interbody fusion which are techniques most spine surgeons are familiar with. Kim and Wu et al. had earlier addressed the safety and efficacy of the ETLIF technique in challenging clinical scenarios of high grade spondylolisthesis [17], severe foraminal stenosis [18], scoliosis [19] and revision [20]. There was no previous study done to evaluate different ways of handling the inferior articular process from inside-out compared to outside-in using ETLIF technique. The details in terms of the direction of endoscopic drilling and start point may not be significant in open or microscopic surgery. However, the direction of endoscopic drilling is important in the uniportal full endoscopic procedure as the direct magnified field with limited view of the isthmus in uniportal endoscope can be disorientating for surgeons who are not familiar with this technique. Often after uniportal endoscope was docked on the target isthmus, soft tissue and in particular multifidus muscle might obstruct the visualization of isthmus as it draped over the isthmus. Dissection of soft tissue towards the interlaminar space often encountered the bleeding rotatores muscle. It can be dangerous if the endoscopic surgeon is disoriented in the interlaminar space as inadvertent drilling into the interlaminar space can lead to dura tear and neural injuries [28].

Clear identification of the start and end point of the endoscopic drilling can promote efficiency in operating time, limit unnecessary soft tissue bleeding and time spent for hemostasis and, most importantly, avoid neural injuries. The ETLIF (O) technique allows early and precise identification under endoscopic vision of most cephalad lateral aspect of inferior articular process intersection with superior articular process (Kim’s point) and subsequent endoscopic drilling in oblique outside-in direction towards the Wu’s point. In ETLIF (I) dissection of multifidus and rotatores spinae in the early part of the surgery is necessary to identify the midpoint of the arc of lamina from the spinolaminar junction and most inferior medial aspect of inferior articular facet (Wu’s point) and endoscopic drilling starts from Wu’s point in the oblique inside-out direction towards Kim’s point. We found that in our cohort of patients, ETLIF (O) achieved statistically significant reduction in operation time for inferior articular process resection and overall total operation time. ETLIF (O) spent a mean 36.55 ± 10.37 min (73.3%) for inferior articular process resection time, 87.45 ± 20.14 min (85.2%) for total operation time required by ETLIF (I), 49.83 ± 23.97 min for inferior articular process resection time and 102.56 ± 36.53 min for total operation time, respectively. We felt that by doing ETLIF (O), we spent less time handling soft tissue dissection and hemostasis of the rotatores spinae muscle which are located medial to Wu’s point. As there is limited muscle mass lateral to the Kim’s point, we could decrease time spent in the early part of surgery dissecting soft tissue as compared to starting at Wu’s point. Being able to identify these key endoscopic anatomical landmarks allowed surgeons to be oriented early in the fusion surgery and decisively start endoscopic drilling of isthmus from outside in direction, which saved time in surgery.

Care must be taken in dissection of both start points. Lateral to Kim’s point in the superficial layer is the superior articular artery which can be controlled by radiofrequency ablation. However, the radicular artery is lying ventral to the intertransverese membrane deep in Kim’s point, and inadvertent damage can lead to significant bleeding and obstruction to endoscopic visualization, sometimes even requiring conversion to microscopic surgery in literature [29]. Medial to Wu’s point is the interlaminar space and inadvertent damage can lead to neural injuries. Identification of key endoscopic landmarks is important in order to decrease the length of the operation, avoid unnecessary bleeding and soft tissue dissection, avoid unnecessary conversion to open surgery due to poor visualization and potentially shorten the learning curve for endoscopic surgeons who are embarking on full facet sacrificing Endoscopic Posterolateral Transforaminal Lumbar Interbody Fusion.

As the subsequent steps in handling of superior articular process, end plate and cage insertion were similar in both ETLIF (I) and ETLIF (O): they both allowed a large cage to be inserted in the intervertebral space. We found both ETLIF approaches to be as proficient in correction of coronal wedge angle, disc height, improving clinical parameters of VAS pain score and ODI score with statistical improvement within each cohort compared to pre and postoperative parameters but no statistical difference was found between the two cohorts. These findings are concordant with earlier studies and meta-analysis [16,19].

Recent metanalysis of endoscopic fusion showed ODI significantly improved by twice as much as the MCID and the VAS for back and leg pain showed significant improvements over the MCID. The perioperative complications were usually minor [16]. We found similar findings within each cohort of ETLIF (I) and ETLIF (O) respectively with improvement in VAS and ODI at all time points of the study. As the disc preparation and superior articular process preparation were similar in both cohorts, there was no statistical difference between the two cohorts, with similar improvements in VAS and ODI. There was no difference in patients’ overall clinical outcome and return to function as both cohorts showed a high percentage of good to excellent outcomes in MacNab’s score.

Despite differences in the handling of IAP, the ETLIF (O) and ETLIF (I) techniques both still have significant technical difficulties in adequate end plate preparation and cage insertion. The limited size of the working channel (6 mm) in uniportal endoscope requires the use of a relatively small endoscopic drill and instruments for disc preparation as compared to open or biportal surgery. The challenge is to ensure endoscopic drilling does not violate the end plate. The clarity of endoscopic vision within the intervertebral disc space can help to decrease the risk of inadvertent damage to the end plate. Fluoroscopic guided cage insertion with subsequent endoscope inspection requires firm handling and an understanding of the position of the retractor tube in relation to the neural element. Both of these technical difficulties have a steep learning curve. We hoped that by highlighting the anatomical landmarks and suggestion of these two ways of approaching the IAP in ETLIF (O) and ETLIF (I), we could help to shorten the learning curve at the first stages of ETLIF.

There was a steep learning curve with higher possible complication rates in the early phase of practice [22]. We found that both ETLIF (O) and ETLIF (I) had low complication rates in our series. Guidance from fluoroscopy and intraoperative navigation could further assist to decrease complications in the earlier cases of ETLIF [30]. We felt that ETLIF in general achieved good direct decompression of the neural elements as compared to uniportal endoscopic transkambin fusion through transforaminal approach (KLIF) which might require a separate additional interlaminar decompression after completion of the fusion procedure and hence poses an additional risk in patients who require such additional decompression. However, there is no study comparing the KLIF approach and ETLIF. No long term study in ETLIF had been performed in literature, however, there were favorable outcomes in various studies of endoscopic decompression compared to open and minimally invasive spinal decompression [31,32,33,34,35] and short to medium term evaluation of ETLIF [17,18,19]. The comparable good clinical outcomes in both cohorts of ETLIF in this study is promising for consideration in using these techniques for fusion.

## 5. Limitations

There are several differences and possible confounding factors in this study: the data was obtained as a retrospective comparative cohort study with patients who had undergone ETLIF (O) and ETLIF (I). There could be inherent selection and performance bias in the study. Pre-operative data such as comorbidities, Charlson Morrison Index, BMI and smoking history were not collected which might introduce confounders in the study. We limited these confounding factors by having the same team of anesthetists and surgeons for both cohorts of operations performed in the data set for both groups. There was a statistically significant different, though not clinically significant, follow up period between the two cohorts due to medium term duration follow up. We continued to follow up on these patients with a view to showing the effect of a longer follow up in the future to evaluate the clinical and radiological data in the long term. Fusion and subsidence evaluation was not performed as this IRB approval did not include the CT scan in our evaluation. A prospective randomized controlled study would be more ideal to eliminate these bias.

## 6. Conclusions

In endoscopic posterolateral transforaminal lumbar interbody fusion, complete removal of Inferior Articular Process is the key part of surgery with two methods that can be applied: an inside-out approach or an outside-in approach. Comparing both techniques, the outside-in approach has a shorter operative time required for inferior articular process resection and a shorter total length of operation with similar good clinical and radiological outcomes.

## Figures and Tables

**Table 1:** Baseline demographics data, radiographic and clinical parameters of Endoscopic Posterolateral Transforaminal Lumbar Interbody Fusion Inside-Out, ETLIF (I) and Endoscopic Posterolateral Transforaminal Lumbar Interbody Fusion Outside-in, ETLIF (O).

	ETLIF (I)	ETLIF (O)	*p* Value
Number of patients	48	38	N.A
Age (mean ± SD in years)	65.02 ± 9.69	68.39 ± 11.41	0.142
F/U Period (mean ± SD in months)	14.73 ± 5.33	11.58 ± 3.81	**0.003**
Operation Time For Inferior Articular Process Resection (mean ± SD in minutes)	49.83 ± 23.97	36.55 ± 10.37	**0.002**
Total Operation Time (mean ± SD min)	102.56 ± 36.53	87.45 ± 20.14	**0.025**
Disc Height Pre-operatively (mean ± SD mm)	7.29 ± 3.07	7.63 ± 3.25	0.615
Disc Height Post-operatively (mean ± SD mm)	12.25 ± 2.74	13.12 ± 1.88	0.097
Coronal Wedging Pre-operatively (mean ± SD°)	5.54 ± 3.75	6.09 ± 5.67	0.591
Coronal Wedging Post-operatively (mean ± SD°)	3.78 ± 2.73	2.85 ± 1.72	0.071
Preoperative VAS (mean ± SD)	7.65 ± 1.25	7.58 ± 1.31	0.809
Postoperative VAS at 1 week (mean ± SD)	3.44 ± 0.85	3.26 ± 0.64	0.297
Postoperative VAS at 3 months (mean ± SD)	2.33 ± 0.88	2.39 ± 0.82	0.742
Postoperative VAS at final follow up (mean ± SD)	1.83 ± 0.86	2.16 ± 0.86	0.085
Preoperative ODI (mean ± SD)	74.38 ± 8.72	73.21 ± 10.62	0.578
Postoperative ODI at 1 week (mean ± SD)	33.50 ± 6.52	32.63 ± 5.44	0.587
Postoperative ODI at 3 months (mean ± SD)	26.25 ± 4.72	27.16 ± 5.31	0.659
Postoperative ODI at final follow up (mean ± SD)	23.81 ± 4.85	25.05 ± 5.52	0.404
Percentage MacNab Good To Excellent (%)	97.9	97.4	0.271

**Table 2:** Clinical and Radiographic parameters of Endoscopic Posterolateral Transforaminal Lumbar Interbody Fusion Inside-Out, ETLIF (I).

ETLIF (I)	Mean	Std. Deviation	*p* Value
VAS improvement at 1 weeks	4.21	1.49	*p* < 0.001
VAS improvement at 3 months	5.31	1.68	*p* < 0.001
VAS improvement at final follow up	5.81	1.61	*p* < 0.001
ODI improvement at 1 weeks	40.88	11.23	*p* < 0.001
ODI improvement at 3 months	48.13	10.86	*p* < 0.001
ODI improvement at final follow up	50.56	10.63	*p* < 0.001
Disc Height Increment (mm)	5.00	2.87	*p* < 0.001
Coronal Wedge Angle Improvement (°)	1.76	1.63	*p* < 0.001

**Table 3:** Clinical and Radiographic parameters of Endoscopic Posterolateral Transforaminal Lumbar Interbody Fusion Outside-in, ETLIF (O).

ETLIF (O)	Mean	Std. Deviation	*p* Value
VAS improvement at 1 weeks	4.32	1.32	*p* < 0.001
VAS improvement at 3 months	5.18	1.47	*p* < 0.001
VAS improvement at final follow up	5.42	1.48	*p* < 0.001
ODI improvement at 1 weeks	40.58	10.17	*p* < 0.001
ODI improvement at 3 months	46.05	10.80	*p* < 0.001
ODI improvement at final follow up	48.16	11.71	*p* < 0.001
Disc Height Increment (mm)	5.49	2.33	*p* < 0.001
Coronal Wedging Improvement (°)	2.87	2.25	*p* < 0.001

**Table 4:** Clinical And Radiographic Parameters Of Endoscopic Posterolateral Transforaminal Lumbar Interbody Fusion Inside-Out, ETLIF (I) versus Endoscopic Posterolateral Transforaminal Lumbar Interbody Fusion Outside-in, ETLIF (O).

Group Charateristics	ETLIF (I) Mean ± SD	ETLIF (O) Mean ± SD	*p* Value
Improvement of VAS at 1 week	4.21 ± 1.49	4.32 ± 1.32	0.727
Improvement of VAS at 3 months	1.10 ± 0.83	0.87 ± 0.67	0.158
Improvement of VAS at final FU	0.50 ± 0.74	0.24 ± 0.59	0.078
Improvement of ODI at 1 week	40.88 ± 11.23	40.58 ± 10.16	0.900
Improvement of ODI at 3 months	7.25 ± 4.68	5.47 ± 4.05	0.067
Improvement of ODI at final FU	2.44 ± 3.48	2.11 ± 3.54	0.663
Change In Disc Height(Postop-Preop) (mean ± SD mm)	5.00 ± 2.87	5.49 ± 2.33	0.394
Change In Cornoal Wedging Angle(Postop-Preop) (mean ± SD°)	1.76 ± 1.63	3.24 ± 2.92	0.072

## Data Availability

Data is available upon request from corresponding author.
